# Age-Dependent Influence of Intrinsic and Extrinsic Motivations on Construction Worker Performance

**DOI:** 10.3390/ijerph18010111

**Published:** 2020-12-26

**Authors:** Nobuki Hashiguchi, Shintaro Sengoku, Yasushi Kubota, Shigeo Kitahara, Yeongjoo Lim, Kota Kodama

**Affiliations:** 1 Graduate School of Technology Management, Ritsumeikan University, Osaka 567-8570, Japan; gr0325kr@ed.ritsumei.ac.jp; 2 School of Environment and Society, Tokyo Institute of Technology, Tokyo 152-8550, Japan; sengoku.s.aa@m.titech.ac.jp; 3 Kumagai Gumi Co. Ltd., Tokyo 162-8557, Japan; yakubota@ku.kumagaigumi.co.jp (Y.K.); skitahar@ku.kumagaigumi.co.jp (S.K.); 4 Faculty of Business Administration, Ritsumeikan University, Osaka 567-8570, Japan; lim40@fc.ritsumei.ac.jp; 5 Center for Research and Education on Drug Discovery, Faculty of Pharmaceutical Sciences, Hokkaido University, Sapporo 060-0808, Japan

**Keywords:** motivation, health risk, age, construction workers, productivity, worker performance

## Abstract

The increasing sophistication and complexity of construction technology have also increased workers’ physical risk and psychological stress. This study examined the relationships between health risks, work motivation, and productivity as perceived by construction workers. A hypothetical model of worker perceptions, and the psychological factors influencing these perceptions, was developed. A total of 324 construction workers at a Japanese construction company participated in the study and were divided into two groups: younger (45 years of age and below) and older adults (46 years of age and above). Data were collected using a questionnaire. The differences between the age groups were analyzed with regard to their perceptions of health risks, motivation, work skills, and productivity. Both younger and older workers were affected by intrinsic motivations and extrinsic motivations, but the effect of these motivations on work performance differed depending on age. Higher health risks are anticipated to affect the work motivation and productivity perceptions of older workers. The proposed model and findings of this study contribute to understanding worker motivations and have important implications for labor management of construction projects. By addressing construction workers’ intrinsic (e.g., interest) and extrinsic (e.g., reward) motivations, it is possible to sustainably improve project productivity.

## 1. Introduction

The construction industry is a significant sector for the economy of any nation; it contributes substantially to the gross domestic product (GDP) and employs a significant portion of the workforce [1,2]. However, hot and humid work environments and long hours of the physical workload at construction sites expose workers to chronic fatigue as well as injury and health risks, which can decrease productivity [3,4]. Construction projects with complex and often fragmented schedules may also be affected by issues that delay progress. In the coming decades, an aging population is going to be one of the most important socioeconomic challenges facing developed countries in Europe and Asia. Therefore, companies must adapt to the changing characteristics of an aging workforce [5]. Particularly in Japan, the rapidly increasing age and consequent retirement of workers are affecting productivity in the construction industry [6,7]. Since construction is labor-intensive, a labor shortage is a significant risk to construction projects. To maintain the workforce, construction companies need to be able to motivate older workers while considering the work environment. Barg et al. [8] investigated peer-reviewed papers over the past five decades, and many researchers have mentioned the importance of increasing worker motivation and communication at the workplace for determining the productivity of construction projects.

Shashank, Supata, Kabin, and Nath [9] identified eight factors that affected productivity at construction projects: manpower, environment, materials, equipment, motivation, safety, management, and quality, according to the knowledge of 108 construction professionals. They concluded that motivation has the strongest impact on productivity. Kazaz, Manisali, and Ulubeyli [10] surveyed 82 companies to identify factors that affect construction productivity in Turkey. They found organizational factors to have the highest effect, about 15% higher than average for other factors and emphasized the role of motivation for increased productivity by knowledge of the subject companies. Worker skills, sociopsychological climate of the team, and worker productivity are related to the quality of work and the effectiveness of management decisions, which, in turn, affect the final company performance [11]. To build an effective human resources system for the improvement in corporate profits, the company management needs to have a clear understanding of the factors that affect productivity, the most important of which are motivators and demotivators [12]. Worker motivation is a top priority for modern enterprises. The willingness of workers to do their work is a key factor for organizational success [13]. Thus, motivation has been a topic of great interest to psychologists and business owners in the past few decades [14]. Moreover, Seifert [15] et al. discuss a program to promote health by increasing a person’s awareness and motivation for action. They describe the potential for a person to minimize physical pain and maximize pleasure through motivation.

In this study, we evaluated health risk by resting heart rate and body mass index (BMI) and investigated the relationship between motivation, skill awareness, and productivity of workers of a wide age group. Previous studies have reported the relationship between worker motivation to work productivity in the construction industry [10,12,16,17,18]. However, few studies have evaluated the impact of motivation on workers’ perception of productivity (i.e., awareness to improve work performance) using a combination of psychological factors seen in work awareness at the workplace and physical health risks.

By investigating previous studies, we have developed a model that determines the relationship among the health condition of workers, work motivation, work skills, and awareness of productivity. In our research, the workers at thirty-eight construction sites of a Japanese construction firm (Kumagai Gumi) were split into two different age ranges; and the relationships between health risk indicators, intrinsic motivation, extrinsic motivation, awareness of work skills, and awareness of team performance were analyzed.

On a practical point, in Japan’s construction industry, in which both older workers and turnover rates are increasing, it will be useful for construction companies to understand the awareness of workers of each age group and manage the workforce accordingly. This approach helps sustain a healthy and rewarding workforce while retaining organizational productivity.

## 2. Theoretical Development

Motivation plays a pivotal role in determining worker productivity. Motivated workers reflect well on their organization and increase the likelihood of it achieving its strategic goals [19]. Although employee motivation has been of great interest for many researchers and business owners over the past decades, it is a complex topic that crosses many disciplinary boundaries, including economics, psychology, organizational development, human resource management, and sociology [20]. A general approach to explaining motivation is the needs-based theory. Some theories that relate to workers’ needs include Maslow’s hierarchy of needs [21], Herzberg’s motivation–hygiene theory [22], and self-determination theory [23]. Examples of needs include physiological needs, esteem needs, adequate compensation, good relationships with others, autonomy, and a sense of achievement or competence. According to Herzberg’s theory, worker satisfaction involves two groups of factors: motivational factor (intrinsic) and maintenance factor (extrinsic) [24]. Intrinsic motivation refers to work done because workers find it inherently enjoyable, interesting, and satisfying [25]. Intrinsic goals include satisfying basic psychological needs, such as personal growth, increased competence, support of others, and relationships with others [26]. Intrinsic motivation is positively correlated with desirable worker outcomes, such as performance and commitment, and negatively correlated with unfavorable outcomes, such as turnover, burnout, and work–family conflict [27]. Extrinsic motivation includes financial success, performance-based incentives, and social recognition. The relationship between intrinsic and extrinsic motivations has been the subject of much research. Some have argued that extrinsic motivation negatively affects intrinsic motivation, while others suggest that they may coexist and be independent of or dependent on each other [28]. However, few studies have described workers’ perception of work performance, taking into account both intrinsic and extrinsic motivations [29].

In this study, we developed hypotheses based on a few motivation theories [22,24,25] and behavioral economics to explain the relationships between intrinsic and extrinsic motivations, each type of motivation and performance, and each type of motivation and other workers. Based on our analysis, we discuss how worker motivation can be managed to improve the productivity of construction projects in a sustainable manner.

### 2.1. Definition of Variables

We developed a hypothetical model of worker perceptions of five latent variables based on the theoretical and empirical research in the literature described previously. The latent variables are explained below.

#### 2.1.1. Health Risk Indicators

In this study, resting heart rate and BMI were used as health risk indicators. Studies have shown that strict management of the working environment is necessary for ensuring worker health at construction sites [30,31,32]. According to Huang and Hsu [33], overwork and job pressure can manifest as physical health risks for workers and affect their motivation. Thus, motivation for physical activity is based on health. Kim and Beehr [34] noted the relationship between worker health and intrinsic motivation. Furthermore, psychological health has also been associated with motivation in the workplace [35] and improved performance [36,37]. The above research indicates that good physical and mental health leads to motivation to work.

#### 2.1.2. Intrinsic Motivation

Intrinsic motivation arises from within an individual. Research has shown that intrinsically motivated workers tend to have higher job satisfaction and performance because they find their work interesting, challenging, and meaningful [36,38]. In addition, intrinsically motivated workers work hard because their work is interesting rather than because of financial rewards [22,25]. Research in the construction industry has shown that workers are intrinsically motivated if their work is challenging and interesting [8,39].

#### 2.1.3. Extrinsic Motivation

Extrinsic motivation originates from factors external to the individual that lead to a specific outcome [25]. Extrinsically motivated people value rewards, recognition from others, promotions, and increased wages. Early studies suggested that extrinsic motivation is more effective at motivating workers than intrinsic motivation [40]. In the construction industry, tangible rewards, such as financial incentives, higher wages, and on-time payment, motivate workers [16,41,42]. However, there is no consensus among previous studies on a definition for work motivation or which type of motivation is predominant. Further research is needed on intrinsic and extrinsic motivations in the construction industry.

#### 2.1.4. Work Skills

Hartman [43] noted that improving and developing skills is central to increased work productivity. Tabassi et al. [44] stated that acquiring knowledge and skills can affect both motivation and performance. Detsimas et al. [45] reported that worker development is essential to improving the overall performance of the workplace, and Khan [46] noted that training can improve motivation and performance.

#### 2.1.5. Team Performance

**Figure 1:**
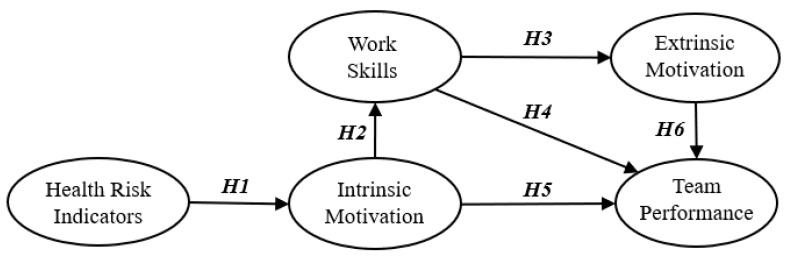
Hypothetical model for the perceptions of construction workers.

Awareness of worker productivity contributes to team effectiveness and organizational performance. Andersson, Rankin, and Diptee [47] showed that collaboration, empathy, and communication between team members are important for team performance. Franco et al. [20] stated that worker performance is influenced by intrinsic and extrinsic motivations as well as work skills. Especially in labor-intensive industries, worker performance depends heavily on motivation. Hornby and Sidney [48] noted that resource availability and worker capacity are essential but not sufficient to ensure acceptable worker performance. Figure 1 shows the hypothetical model for the relationships among the latent variables.

#### 2.1.6. Worker Age

Regarding worker productivity loss due to aging, it has been reported that older workers have fewer accidents and injuries [49,50]. As for those factors, it is presumed that the awareness and experience of older workers allow them to maintain safe and solid work [8,50]. In addition, older workers are reported to adhere to rules and procedures at work [51]. A review by the UK Department of Trade and Industry provided evidence that the physical and mental decline associated with normal aging has little effect on the performance of many jobs, except for jobs requiring fast physical reactions and physical strength [52]. This study shows that the brains of older people might have different functions than the brains of younger people. However, it is not always accompanied by a decline in function [53,54].

Crowfold et al. [55] found that both physical and psychological changes occur in middle-aged and older workers over the age of fifty. However, these changes vary widely among individuals and can be moderated by lifestyle modifications. Therefore, there was no finding of inferior labor productivity with increasing worker age. Dalen et al. [56] defined that hard qualities (e.g., flexibility, physical and psychological abilities, willingness to learn new skills) and soft qualities (e.g., commitment to the organization, reliability, and social skills) in workers’ job skills. It is reported that older people have the advantage of soft qualities and younger people have the advantage of hard qualities. In general, there is a negative stereotypical evaluation of the productivity of older workers. However, few reports have investigated the effects of worker age on work performance regarding productivity, including work awareness. In this research, the above latent variables (health risk indicators, intrinsic and extrinsic motivation, work skills, and team productivity) were used to analyze work awareness by worker age.

### 2.2. Definition of Hypothetical Model

Based on some literature reviews, we hypothesized the following relationships between worker health, motivation, skills, and performance.

#### 2.2.1. Health Risk Indicators and Intrinsic Motivation

Cooney et al. [57] noted that workers in challenging environments are concerned about their health. Worker perception of health risks can lead to higher sensitivity in terms of working conditions and positive behavior (e.g., moral activities and challenging activities). As part of their annual physical examinations, construction companies have begun regularly measuring workers’ physical health measures, such as blood pressure, heart rate, height, and weight, to raise awareness of worker health [58]. Increased resting heart rate has been reported to raise the risk of death and increase cardiovascular and heart disease [59,60]. In a study on East Asians, Zheng et al. [61] found that people with a BMI of 22.6–27.5 had the lowest risk of death from cardiovascular disease, cancer, and other causes; this risk increases with the increase in BMI. In the construction industry, strict work schedules and irregular working hours and temperatures need to be taken into account to ensure workplace safety. Love and Edwards [30] noted that these conditions cause stress in workers and lead to poor performance. Research has shown that strict control of the work environment is necessary to manage worker health at construction sites [30,31,32]. Overwork and work pressure can lead to physical health risks and affect intrinsic motivation. Therefore, Yperen, Wortler, and Jonge [62] argued that companies and managers should consider workers’ perceptions regarding workplace conditions. Huang and Hsu [33] reported that the motivation for physical activity depends on health. Similarly, Cuberos et al. [63] associated a preference for healthy habits with high intrinsic motivation, and Kim and Beehr [34] identified a relationship between worker health and intrinsic motivation. Dagenais-Desmarais, Leclerc, and Londei-Shortall [35] associated psychological health with worker motivation. Psychological health has been reported to be positively and significantly associated with intrinsic motivation and performance [36,37]. Furthermore, physical health and mental health are related [64], and Gerber and Puehse [36] found a positive association between mental health and physical activity. Worker motivation has been associated with both physical and mental health.

**Hypothesis** **1** **(H1).** *Health risk indicators affect intrinsic motivation. (Health risk indicators → Intrinsic motivation).*

#### 2.2.2. Intrinsic Motivation and Work Skills

Bos, Donders, Bouwman-Brouwer, and van der Gulden [65] found that discrepancies between actual work skills and job requirements may negatively impact work life. Matching workers’ expectations with the reality of the workplace can affect motivation. Kuranchie-Mensah and Amponsah-Tawiah [66] showed that a company needs to explain its goals to the workers to motivate them and use their skills to achieve the said goals. Worker motivation is related to their responsibilities, and the different needs and motivations of individual workers need to be considered. Cardoso, Dominguez, and Paiva [67] argued that feedback on developmental requirements, such as autonomy at work, participation in setting organizational goals, education, and training, can improve worker skills and performance. Feibel, Peter, Swart, and March [68] stated that meaningful, responsible, and essential work suitable to the workers’ age should be assigned according to their physical and mental abilities. Kase, Saksida, and Miheli [69] noted that during skill development, motivation is enhanced by the learner’s perception of their growth. For older learners, skill development is mostly driven by intrinsic motivation. Kaufmann and Schulze [70] showed that motivation can affect a worker’s skill set when they enjoy solving work challenges.

**Hypothesis** **2** **(H2).** *Intrinsic motivation and work skills. (Intrinsic motivation → Work skills).*

#### 2.2.3. Work Skills and Extrinsic Motivation

Improving skills at the workplace, such as technical competence and equipment proficiency, is core to improving workers’ performance. Hartman [44] stated that the motivation to innovate and improve productivity requires advanced skills to solve project-related problems. Kanungo and Mendonca [71] noted that much of the conceptual framework for worker motivation had been established in developed countries. Franco et al. [20] reported that workers’ extrinsic motivations have also been supported by research in developing countries, along with their relationship with work skills. Sayers [72] pointed out that some workers rely heavily on their skills and judgment when working independently and are motivated by the desire to improve their professional skills and increase their value for future employers. Research indicates that workers focus on career self-development value, increasing their professional skills to improve their prospects for higher salaries and personnel evaluations [72,73,74]. Hammill [75] showed that a worker’s decision to stay in or leave a company depends on the opportunity for professional development and a preference for direct and immediate assessment and reward. Dougan et al. [73] argued that such workers are less tolerant of waiting for their turn to be promoted; hence, they should be readily recognized and rewarded for a good job.

**Hypothesis** **3** **(H3).** *Work skills affect extrinsic motivation. (Work skills → Extrinsic motivation).*

#### 2.2.4. Work Skills and Team Performance

The acquisition of knowledge and skills has been shown to affect work performance and motivation [45,46]. Mittal, Dhiman, and Lamba [76] provided an example of how improving the skills of blue-collar workers resulted in improved quality and reduced defects. Khan [47] noted that performance can be improved by training workers to enhance their skills. Improvement in work skills is central to increasing worker productivity [77]. Abiodun and Kanda [78] quantified the decline in worker productivity due to inadequate work skills. According to Vänni, Virtanen, Luukkaala, and Nygård [79], a 1% reduction in work capacity can result in a 5% reduction in worker productivity.

**Hypothesis** **4** **(H4).** *Work skills affect team performance. (Work skills → Team performance).*

#### 2.2.5. Intrinsic Motivation and Team Performance

Company performance is influenced by the motivation of its workers. Turner [80] noted that understanding whether worker motivation is intrinsic or non-intrinsic can help a company identify the drivers of its performance. Previous research on construction companies has indicated that training can improve motivation, which, in turn, can improve efficiency and teamwork. Tabassi et al. [45] showed that with organized development, intrinsic motivation can have a significant impact on worker performance. In many workplaces, worker motivation has been shown to have a positive and significant relationship with performance [37,81]. According to Shahzadi et al. [81], when workers are intrinsically motivated, they perform at relatively high levels because they have an interest in and derive enjoyment from the work. Although intrinsic motivation is useful for achieving high performance, many studies have focused on extrinsic motivation, even in developing countries, where labor compensation is relatively low [82].

**Hypothesis** **5** **(H5).** *Intrinsic motivation affects team performance. (Intrinsic motivation → Team performance).*

#### 2.2.6. Extrinsic Motivation and Team Performance

Extrinsic motivation has a significant impact on worker productivity in the construction industry, especially in developing countries where companies are motivated by materially favorable conditions [42]. Research has shown that contractors tend to prefer non-monetary incentives, but workers are primarily motivated by monetary rewards (high salaries, bonuses, opportunities for promotion, recognition for effort, and job security) [16,42]. According to Turner [80], extrinsic motivation is used to entice workers; therefore, identifying factors that extrinsically motivate workers is useful for companies. Research has shown that organizational leaders or managers perceive worker motivation as a complex system and recognize the importance of intrinsic incentives, but workers perceive extrinsic rewards as stronger motivators [17,42,43]. Rewards are not the only type of extrinsic motivation that significantly affect performance. Aydin [83] found that performance appraisals also have a significant impact on and can affect work productivity. Yoon, Sung, Choi, Lee, and Kim [84] found a positive correlation of worker creativity/improvement with extrinsic motivation (evaluation by supervisors and others), but not with intrinsic motivation.

**Hypothesis** **6** **(H6).** *Extrinsic motivation affects team performance. (Extrinsic motivation → Team performance)*

## 3. Methodology

### 3.1. Construction Workers’ Model and Hypothetical Relationships

A questionnaire was used to investigate the associations between productivity, team performance, health risks, motivation, and employee skills within the target construction company. The intrinsic and extrinsic motivations of the workers were evaluated with Spector’s [85] Job Satisfaction Survey. Extrinsic motivation included promotion and salary. In total, seven items were used to evaluate extrinsic motivation: four related to promotions and three related to salary. Conversely, intrinsic motivation included the sub-type of work and supervision. In total, eight items were used to evaluate intrinsic motivation: four for each sub-type. Workers’ perceptions of work skills and workplace productivity were assessed by the Work Safety Scale (WSS) [86]. Worker awareness of the workplace, supervisor–worker relationship, job satisfaction, job attitudes, and skills were measured from the 2011 Workplace Employment Relations Survey questions [87,88].

After interviewing experienced site supervisors within the target construction firm, a questionnaire consisting of psychological factors that are thought to influence construction workers’ motivation was devised. The questionnaire comprised five items related to the work environment. All items were measured on a five-point Likert scale (1 = *strongly disagree*, 5 = *strongly agree*). We used the self-developed questionnaire to assess the workers’ perception of the work environment.

### 3.2. Protocol

The purpose of the study was first explained to all participants, and then the questionnaire was administered to all participants. Their rights were also explained: participation would not cause any disadvantage to them, the collected data would be anonymous, and participants could refuse to answer the questionnaire. In compliance with the Universal Declaration of Human Rights, the Declaration of Helsinki, and the Human Genome Project, the protocols for collecting personal information about the age, heart rate, physical characteristics, and consciousness of the participants we deal with in our research have been approved by the Ethics Committee of Ritsumeikan University. To minimize the risk of revealing personal information, the names of the participants in the survey were not used during the course of the analysis; instead, each participant was assigned a personal identification code.

### 3.3. Participants

The study was conducted in 2018. In the questionnaire survey, workers of a wide range of ages were selected by convenience sampling at 38 construction sites of a Japanese construction company (Kumagai Gumi, where the co-researchers belong). In this survey, a total of 25 workers at the two construction sites declined the questionnaire. In addition, the 33 participants who did not answer any one or more of the survey items were excluded from the analysis. A total of 382 questionnaires were distributed, for a response rate of 84.8% (valid response rate = 93.5%). In total, 324 participants were included in the analysis and divided into two groups based on the average age of the sample: (A) 165 young workers (45 years old and below) and (B) 159 older workers (46 years old and above). Table 1 presents the demographic information of the subjects, including their age, gender, experience, employment status, BMI, resting heart rate, and group composition.

### 3.4. Data Collection

Data were collected from participants’ questionnaire responses regarding resting heart rate, height, weight, and work awareness. The questionnaire asked for physical information on height and weight, biometric information on resting heart rate, and workers’ awareness. In the targeted construction companies, workers had regular physical examinations so that they could learn about their physical health measures. In the targeted construction company, workers underwent regular physical examinations so that they would have access to their physical health measures. Responses to the questionnaire were assessed using a five-point Likert scale to assess the impact of participants’ awareness on latent variables [89,90]. Participants were asked to answer questions about intrinsic and extrinsic motivation, awareness of work skills, and team performance (Appendix A).

### 3.5. Data Analysis

The subjects’ questionnaire responses were analyzed by structural equation modelling (SEM). First, confirmatory factor analysis (CFA) was used to analyze the relationships between the latent variables observed in the hypothetical model. The programming language for statistical analysis was R language 3.5.1. We compiled the questionnaire results and performed statistical analyses on Groups A and B. Before the SEM analysis of the questionnaire responses, CFA was used to assess the reliability of the responses, where the correlation between the hypothetical model and survey data was tested [49]. In the hypothesis model, the observed variables with weak factor loadings of observed variables were removed [91]. Groups A and B initially had 19 observational variables, but the CFA showed that nine observational variables did not have strong factor loadings of latent variables in both groups. These were eliminated, resulting in 10 variables [92]. The removed variables were WIM4, WS4, WEM3, WEM4, WEM5, WEM6, TP4, and TP5 (see Appendix A). Then, the measurement data were evaluated, and the reliability and validity of the model were confirmed [91]. The reliability and validity of the obtained data were confirmed by Cronbach’s alpha [93]. In general, values greater than 0.7 indicate sufficient data reliability [94]. Table 2 lists the reliability values for all constructs. All values exceeded 0.7 in both groups, indicating the reliability of the target dataset. Therefore, the dataset was used for analysis.

The fitness of the models in both groups was tested based on the goodness of fit (GoF). The GoF was assessed according to the Normkai square (X2/df), comparative fit index (CFI), Tucker–Lewis index (TLI), goodness of fit index (GFI), and root mean square error of approximation (RMSEA). The GoF indices for both models were obtained according to the approach that is recommended by Yuan et al. [18]. The GoF indices for both groups fit the recommended values, as given in Table 3. Thus, the hypothetical model showed a good fit for both groups.

## 4. Results

Using BMI and resting heart rate as observed variables, the effects of the health risk index (HRI) as a latent variable were examined. The SEM used in this study deals with linear relationships. SEM was used to analyze the relationship between HRI and the other four latent variables. The percentages of worker responses obtained by the questionnaire are summarized in Appendix B for each of the younger and older worker groups. In both groups, the latent variables of work skills (WS), intrinsic motivation (WIM), extrinsic motivation (WEM), and team performance (TP) had a high percentage of Neutral responses and tended to exist between Neutral and Strongly agree. Table 4 presents the descriptive statistics and correlation outcomes for Group A. The latent variable with the highest score was team performance (TP) with a mean (standard deviation, *SD*) of 3.93 (*0.910*), followed by intrinsic motivation (WIM) with a mean (*SD*) of 3.92 (*0.866*) and extrinsic motivation (WEM) with a mean of 3.61 (*0.988*). Work skills (WS) had a mean score of 3.20. HRI showed no statistically significant correlation with other latent variables. All of the remaining latent variables were positively correlated to each other. By indicating the correlation coefficient in r and the *p*-value in *p*, the correlations were seen between WIM and TP (r = 0.686, *p* < 0.001), WEM and TP (r = 0.533, *p* < 0.001), and WIM and WEM (r = 0.476, *p* < 0.001). The correlations were weakest between WS and TP (r = 0.321, *p* < 0.001), WS and WIM (r = 0.179, *p* < 0.05), and WEM and WS (r = 0.166, *p* < 0.05). The strongest correlation was between WIM and TP

Table 5 presents the descriptive statistics and correlation results for Group B. The latent variable with the highest score was TP with a mean (*SD*) of 4.10 (*0.841*), followed by WIM with a mean (*SD*) of 3.95 (*0.761*) and WEM with a mean (*SD*) of 3.71 (*0.967*). This is similar to the results for Group A. WS had a mean score of 3.15. HRI showed negative correlations with WIM, WEM, and TP; however, the correlations with WEM and TP were not significant. The positive correlations between other latent variables and HRI were also not significant. All correlations between the other latent variables were positive and significant. The largest correlation coefficients between WIM and TP (r = 0.541, *p* < 0.001), WEM and TP (r = 0.579, *p* < 0.001), and WIM and WEM (r = 0.416, *p* < 0.001). The smallest correlation coefficients were between WIM and WS (r = 0.227, *p* < 0.01), WS and TP (r = 0.332, *p* < 0.001), and WEM and WS (r = 0.178, *p* < 0.05).

**Figure 2:**
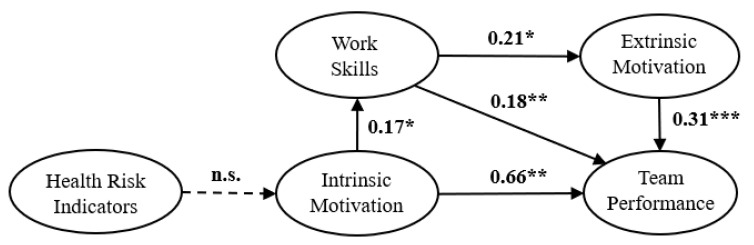
The standardized parameter estimates in the final model for Group A. *: *p* < 0.05; **: *p* < 0.01; ***: *p* < 0.001; n.s.: not significant. The path coefficients are expressed in standardized estimates.

**Figure 3:**
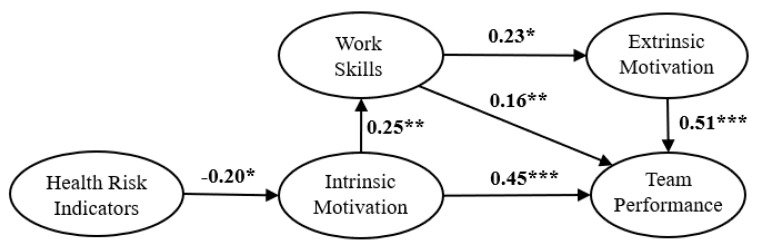
The standardized parameter estimates in the final model for Group B. *: *p* < 0.05; **: *p* < 0.01; ***: *p* < 0.001. The path coefficients are expressed in standardized estimates.

SEM was used to test the reliability and validity of the hypothetical model. SEM is an excellent statistical approach for understanding social and natural phenomena by examining the statistical relationship between latent variables and observable variables. The hypothesis of causality between variables can be tested by observed data, such as questionnaire responses, test scores, and experimental results. We used regression analysis and path analysis to estimate the strength and direction of the hypothesized causal relationships as well as to validate the model. This approach does not account for measurement errors; the regression and path analyses were required to confirm that the model assumptions are valid. Figure 2 and Figure 3 show the derived path diagrams of Groups A and B with standardized solutions for the strengths of the path coefficients [93]. SEM analysis using the R language provides the significance of the path in the relationship between the latent variables [95,96], in addition to the path estimates and standard deviation. The p-values regarding the path coefficients between latent variables in each group are shown in Figure 2 and Figure 3.

Groups A and B were analyzed independently to examine the relationships among the latent variables. The direct and indirect effects of the latent variables in the model were determined. The path coefficients in the hypothetical models represent estimates of the standardized parameters. For the Group A model in Figure 2, H1 was not statistically significant, but the rest of the hypotheses were supported. For Group B in Figure 3, H1 was in the negative direction, while the other hypotheses were in the positive direction. The path coefficients were significant, and all hypotheses were confirmed for Group B.

The hypothetical model assumes that HRI directly affects WIM and indirectly affects TP. The results indicated that WIM, WEM, and WS directly affect TP regardless of worker age and that HRI indirectly affects TP for older workers.

Both groups were analyzed to define the relationship between the predicted direction and latent variables. The direct and indirect effects of the latent variables in the model were determined, and the results are shown in Table 6. The results suggested that an increased HRI for older workers negatively affects WIM; this caused WIM, WEM, and WS to affect TP negatively. These relationships were confirmed only for Group B. For Group A, HRI had no significant effect on the other latent variables. Thus, HRI influenced the perception of TP only among older workers.

## 5. Discussion

### 5.1. The Status of the Construction Industry in Japan

The numbers of Japanese construction workers peaked at 6.85 million in 1997 but fell to 5.30 million in 2018. The majority of construction workers are middle-aged and older; 34.1% are over 55 years old, and only 11.0% are below 29 years old. It is necessary to ensure long-term leadership to pass on the technology to the next generation [97,98]. In this study, we investigated worker motivation at a construction company in 2018. A two-sample Kolmogorov–Smirnov test (two-tailed *p* = 0.8928) was used to examine whether the two sample groups were equivalent for age. Table 7 shows the age composition of our study participants and Japanese construction workers; the difference is less than 5% of the significance level. This indicates that the age distribution of the study participants was similar to that of Japanese construction workers.

### 5.2. Construction Workers’ Motivation

Early research indicated that extrinsic motivation is sufficient for improving work performance. However, Mickel, and Barron [40] reported that intrinsic motivation is effective for tasks that are more complex and require higher cognitive skills. Extrinsic motivation has been reported to contribute more than intrinsic motivation for the performance of relatively simple jobs in labor-intensive industries [99,100]. Our results confirmed that both intrinsic and extrinsic motivations significantly affect the performance of construction workers in terms of awareness. Construction workers perform cognitive skill-intensive tasks; hence, both intrinsic motivation and extrinsic motivation affect their engagement. This result is consistent with previous research by Putra, Cho, and Liu [101]. In contrast to the crowding theory, which claims that extrinsic motivation reduces intrinsic motivation [102,103], our results supported previous studies reporting extrinsic motivation does not reduce intrinsic motivation [101,104,105].

### 5.3. Construction Workers’ Health Risk Indicators and Perceptions

Intrinsic motivation, extrinsic motivation, and awareness of work skills differed in their effects on the perception of work performance for each age group. Intrinsic motivation had a greater impact on the awareness of work performance for younger workers than for older workers. Extrinsic motivation had a greater impact on the awareness of work performance for older workers. For both age groups, intrinsic motivation affected their perception of their work skills, and workers with high intrinsic and extrinsic motivations tended to have a greater awareness of their work performance. The effect of motivation on perceived performance demonstrated in the present study is consistent with previous research [37,75,79,80]. Nyambegera and Gichery [106] reported that intrinsic motivation affects improvement in work skills. However, our results confirmed that work skills are stimulated by extrinsic motivation, which is supported by prior research [37,45,46,47,76]. Our results suggested that health risk indicators did not significantly affect the other latent variables for younger workers. Thus, H1 was not supported while the other hypotheses of the hypothetical model were confirmed. In contrast, health risk indicators had a negative effect on intrinsic motivation for older workers and indirectly affected the other latent variables. For the older workers, increased health risk was accompanied by a decline in intrinsic motivation, which suggested a decrease in work skills and awareness of work performance via extrinsic motivation.

### 5.4. Future Prospects for Understanding Construction Workers

Worker motivation is a complex and dynamic process [20]. The framework of this study could be applied to assessing worker motivations and their perception of construction projects. Since construction projects involve several day-to-day business issues, labor management is critical to ensure sustainable development in the construction industry. Construction companies need to manage the various skills, experiences, and motivations of their workers as the project progresses; this is important since the number and quality of workers required on a construction site changes as the project progresses.

Kooji et al. [107] noted that work-related growth motivation (intrinsic motivation) decreases with increasing age. Contrarily, Rhodes [108] reported that the desire for safety and belonging (extrinsic motivation) increased with age, while the intensity of self-actualization and growth motivation (intrinsic motivation) decreased. These studies are consistent with our results. Younger workers tended to focus on intrinsic motivation for their perception of work performance. Thus, their motivation can be increased by shifting them from mundane and simple duties to more difficult and meaningful tasks. Meanwhile, older workers were predominantly affected by extrinsic motivation in their perception of work performance. For them, intrinsic motivation can help with the development of new skills [109,110]. Continuous learning can help increase the intrinsic motivation of older workers [69].

### 5.5. Theoretical and Practical Implications

The results of this study can be useful for the labor management of workers in different age groups. Construction workers are affected by both intrinsic and extrinsic motivations, which differ with respect to their effect on the perceived work performance depending on the age group. Increased health risks may reduce the intrinsic motivation and perceived work performance of older workers. However, increased health risk does not seem to affect the intrinsic motivation and perceived work performance of younger workers. Several previous studies on worker motivation have used interviews with construction workers [10,12,17,18,45,46,111]. However, few included biometric and physical characteristics in their analysis. To the best of our understanding, this research is the first to analyze the relationship among health awareness, motivation, and productivity in construction workers. Our study provides important information on the motivations of workers for construction companies, especially small- and medium-sized enterprises that are struggling to deal with an aging and retiring workforce. Further research can contribute to the management of construction workers in terms of sustaining and enhancing their motivation and productivity as they age.

### 5.6. Limitations

This study has several limitations. First, the survey data focused on a wide range of worker ages in a Japanese construction company and inferred a causal relationship between workers’ perceptions and health status. The organizational activities at production sites in Japan are characterized by continuous improvement through collective activities, positive attitudes towards quality and safety, and high levels of worker empowerment [112,113]. In future research, it may be necessary to investigate the impact of organizational culture, job engagement, and job craft behavior on more construction workers. Second, we used a self-reporting method, which may have resulted in differences in workers’ resting heart rate, and BMI. Future studies should strive to include actual observed data to identify their potential impact on workers. Third, workers’ awareness is complex and influenced by their experience, physical characteristics, and personal circumstances in addition to their age; this requires further research. More evidence is needed to confirm the accuracy of the hypothesis model, and further research is needed in other work environments. Finally, the hypotheses in this study were wide-ranging, with many side effects and adverse effects as well as endogenous problems. In addition, as this study mainly focuses on a specific group, further research and discussion are needed to determine whether it can be promoted to more groups in the future. These results may be specific to the Japanese construction company workers in this study and need to be evaluated for generality.

## 6. Conclusions

This study was conducted to assess the productivity and motivation of workers in a Japanese construction company by categorizing them into two age groups. For older workers, increased health risk had a negative impact on intrinsic motivation and affected their perceived work performance, work skills, and extrinsic motivation. For younger workers, increased health risk did not affect intrinsic motivation or perceived work performance. Intrinsic and extrinsic motivations had a positive effect on perceived work productivity for all construction workers. Younger workers placed more emphasis on intrinsic motivation, while older workers placed more importance on extrinsic motivation. Regular monitoring of biometric and physical health information, such as heart rate and BMI, can help construction companies understand the perceptions of construction workers and promote a sustainable relationship with their labor force [114]. Quantifying health risks and motivation can improve work quality and worker health. Furthermore, understanding the perceptions of young workers can help avoid the risk of mid-career retirement.

## Figures and Tables

**Table 1:** The demographic of the participants.

Group	Total	%	Category		Number
Young workers (Group A)	165	50.9	Age (years old)	≤25	21
				26–35	60
				36–45	84
			Gender	Male	163
				Female	2
			Experience (years)	≤10.0	81
				10.1–20.0	59
				20.1–30.0	25
				30.1–40.0	0
				≥40.1	0
			Employment level	Worker/Helper	4
				Engineer/Technician	141
				Supervisor/Manager	20
			BMI	≤22.5	63
				22.6–27.5	79
				≥27.6	23
			Resting heart rate (beats/min)	≤69	52
				70–79	83
				80–89	29
				90–99	1
				≥100	0
Older workers (Group B)	159	49.1	Age (years old)	46–55	95
				56–65	46
				≥66	18
			Gender	Male	159
				Female	0
			Experience (years)	≤10.0	32
				10.1–20.0	27
				20.1–30.0	58
				30.1–40.0	30
				≥40.1	12
			Employment level	Worker/Helper	11
				Engineer/Technician	122
				Supervisor/Manager	27
			BMI	≤22.5	58
				22.6–27.5	74
				≥27.6	27
			Resting heart rate (beats/min)	≤69	0
				70–79	46
				80–89	90
				90–99	23
				≥100	0

Note: BMII = Body mass index.

**Table 2:** Validity and reliability of the test results in the two groups of workers.

Latent Variables	Group A				Group B			
	Initial		Final		Initial		Final	
	Items	Alpha	Items	Alpha	Items	Alpha	Items	Alpha
Questionnaire	19	0.896	10	0.867	19	0.873	10	0.865
Intrinsic motivation	4	0.824	3	0.893	4	0.785	3	0.896
Work skills	4	0.752	3	0.919	4	0.756	3	0.861
Extrinsic motivation	6	0.674	2	0.882	6	0.708	2	0.790
Team performance	5	0.838	3	0.876	5	0.761	3	0.811

**Table 3:** Validity of the models for Groups A and B, and recommended goodness of fit (GoF) values.

Fit Indices	Group A		Group B		Recommended Value [18]
	Initial	Final	Initial	Final	
χ^2^/df	3.39	2.15	2.91	1.79	From 1–5
CFI	0.769	0.954	0.734	0.950	≒1
TLI	0.724	0.936	0.738	0.934	≒1
GFI	0.980	0.994	0.984	0.996	≒1
RMSEA	0.121	0.084	0.108	0.071	<0.05 indicates very good fit (threshold level = 0.1)

Note: CFI = comparative fit index; TLI = Tucker–Lewis index; GFI = Goodness of fit Index; RMSEA = Root mean square error of approximation.

**Table 4:** Correlation matrix and descriptive statistics for the young workers’ group (Group A).

Latent Variables	Mean	SD	HRI	WIM	WS	WEM	TP
Health risk indicators	-	-	1.00				
Intrinsic motivation	3.92	0.866	0.013	1.00			
Work skills	3.20	0.911	0.110	0.179 *	1.00		
Extrinsic motivation	3.61	0.988	−0.076	0.476 ***	0.166 *	1.00	
Team performance	3.93	0.910	−0.063	0.686 ***	0.321 ***	0.533 ***	1.00

Note: HRI = Health risk indicators; WIM = Intrinsic motivation; WS = Work skills; WEM = Extrinsic motivation; TP = Team performance; SD = standard deviation. * indicates *p* < 0.05; *** indicates *p* < 0.001.

**Table 5:** Correlation matrix and descriptive statistics for the older workers’ group (Group B).

Variables	Mean	SD	HRI	WIM	WS	WEM	TP
Health risk indicators	-	-	1.00				
Intrinsic motivation	3.95	0.761	−0.157 *	1.00			
Work skills	3.15	0.823	0.005	0.227 **	1.00		
Extrinsic motivation	3.71	0.967	−0.068	0.416 ***	0.178 *	1.00	
Team performance	4.10	0.841	−0.048	0.541 ***	0.332 ***	0.579 ***	1.00

Note: HRI = Health risk indicators; WIM = Intrinsic motivation; WS = Work skills; WEM = Extrinsic motivation; TP = Team performance; SD = standard deviation. * indicates *p* < 0.05; ** indicates *p* < 0.01; *** indicates *p* < 0.001.

**Table 6:** The summary of direct, indirect, and total effects of path.

Path Direction	Group A			Group B		
	Direct	Indirect	Total	Direct	Indirect	Total
HRI → WIM	n.s.	-	n.s.	−0.20	-	−0.20
WIM → TP	0.66	0.04	0.70	0.45	0.07	0.52
WEM → TP	0.31	-	0.31	0.51		0.51
HRI → TP *	n.s.	-	n.s.	-	-	−0.11

Note: HRI = Health risk indicators; WIM = Intrinsic motivation; TP = Team performance; WEM = Extrinsic motivation. n.s.: not significant.; *: The total effect of HRI → TP was determined by multiplying the path coefficients of HRI → WIM and WIM **→** TP. Thus, in Group A, HRI → TP was not significant, and in Group B, HRI → TP = −0.11 was obtained. The regression in group B were WIM = −0.20 × HRI and TP = 0.52 × WIM.

**Table 7:** The distribution of age groups among the participants and Japanese construction workers.

Age (Years Old)	Participants		Japanese Construction Workers	
	Number	%	Number (×10^4^)	%
≤25	23	6.4	20	6.7
26–35	63	17.6	43	14.4
36–45	97	27.2	67	22.5
46–55	106	29.7	68	22.8
56–65	48	13.4	56	18.8
≥66	20	5.6	44	14.8
Total number	357	100.0	298	100.0
Average age (years old)	44.8		45.6	

## Data Availability

All relevant data are within the paper. The data underlying this study are the surveyed company’s data and are available to all researchers on reasonable request from the corresponding author.

## Appendix A

**Table A1:** Survey on psychological factors regarding motivation and awareness of work.

	Questionnaire Items	Strongly Disagree	Disagree	Neutral	Agree	Strongly Agree
WS1	I have technical skills for work.	^1^□	^2^□	^3^□	^4^□	^5^□
WS2	I am skilled in using devices and tools for work.	^1^□	^2^□	^3^□	^4^□	^5^□
WS3	I have work abilities.	^1^□	^2^□	^3^□	^4^□	^5^□
WS4	I have work experience.	^1^□	^2^□	^3^□	^4^□	^5^□
WIM1	I have fun doing my work.	^1^□	^2^□	^3^□	^4^□	^5^□
WIM2	I enjoy this work very much.	^1^□	^2^□	^3^□	^4^□	^5^□
WIM3	I am proud of my work.	^1^□	^2^□	^3^□	^4^□	^5^□
WIM4	I am willing to work.	^1^□	^2^□	^3^□	^4^□	^5^□
WEM1	I have an opportunity for promotion.	^1^□	^2^□	^3^□	^4^□	^5^□
WEM2	I am evaluated by my supervisor.	^1^□	^2^□	^3^□	^4^□	^5^□
WEM3	I am satisfied with my income.	^1^□	^2^□	^3^□	^4^□	^5^□
WEM4	I am satisfied with the safety of the workplace.	^1^□	^2^□	^3^□	^4^□	^5^□
WEM5	I am satisfied with the pressure at work.	^1^□	^2^□	^3^□	^4^□	^5^□
WEM6	I am satisfied with the planned work.	^1^□	^2^□	^3^□	^4^□	^5^□
TP1	Support from my supervisor and co-workers is important.	^1^□	^2^□	^3^□	^4^□	^5^□
TP2	Empathizing with co-workers is important.	^1^□	^2^□	^3^□	^4^□	^5^□
TP3	Collaboration with co-workers is important.	^1^□	^2^□	^3^□	^4^□	^5^□
TP4	Communication with co-workers is important.	^1^□	^2^□	^3^□	^4^□	^5^□
TP5	I am working on a deadline.	^1^□	^2^□	^3^□	^4^□	^5^□

Note: Work skills include WS1, WS2, WS3, and WS4. Intrinsic motivation includes WIM1, WIM2, WIM3, and WIM4. Extrinsic motivation includes WEM1, WEM2, WEM3, WEM4, WEM5, and WEM6. Team performance includes TP1, TP2, TP3, TP4, and TP5.

## Appendix B

**Table A2:** Responses to latent variables for the young workers’ group (Group A).

Latent Variables	Observational Variables	Strongly Disagree (%)	Disagree (%)	Neutral (%)	Agree (%)	Strongly Agree (%)
WS	WS1, WS2	4.5	12.7	53.3	22.2	7.3
WIM	WIM1–WS3	0.0	1.0	35.0	32.5	31.5
WEM	WEM1, WEM2	2.7	4.6	42.1	20.6	30.0
TP	TP1–TP3	0.3	1.2	29.7	25.8	43.0

Note: WS = Work skills; WIM = Intrinsic motivation; WEM = Extrinsic motivation; TP = Team performance.

**Table A3:** Responses to latent variables for the older workers’ group (Group B).

Latent Variables	Observational Variables	Strongly Disagree (%)	Disagree (%)	Neutral (%)	Agree (%)	Strongly Agree (%)
WS	WS1, WS2	1.3	2.5	56.8	26.2	13.2
WIM	WIM1–WS3	0.0	2.3	36.9	37.5	23.3
WEM	WEM1, WEM2	0.9	2.5	42.5	28.0	26.1
TP	TP1–TP3	0.3	1.6	38.4	30.5	29.2

Note: WS = Work skills; WIM = Intrinsic motivation; WEM = Extrinsic motivation; TP = Team performance.
